# HSP90 facilitates stemness and enhances glycolysis in glioma cells

**DOI:** 10.1186/s12883-022-02924-7

**Published:** 2022-11-11

**Authors:** Xun Kang, Jing Chen, Jian-feng Hou

**Affiliations:** 1 Department of Neuro-oncology, Cancer Center, Beijing Tiantan Hospital, Capital Medical University, No.119, South Sihuan West Road, Beijing, 100070 China; 2 Department of Oncology, Luoyang Central Hospital Affiliated to Zhengzhou University, Luoyang, 471000 Henan China

**Keywords:** HSP90, Stemness, Glycolysis, Glioma

## Abstract

**Background:**

Glioma is one of the most commonly occurring malignant brain cancers with high recurrence and mortality. Glioma stem cells (SCs) are a rare sub-group of glioma cells that play a critical role in tumor progression. Heat shock protein 90 (HSP90) is known to promote the stemness of glioma SCs. Here, we investigated the role of HSP90 in glioma SC metabolism, to reveal its potential as a novel therapeutic target.

**Methods:**

Self-renewal assays were used to assess stemness. Cell migration, invasion and viability were measured using Transwell and CCK-8 assays, respectively. Tumor growth was evaluated in xenograft nude mouse models. The expression of known markers of stemness including CD44, A2B5, Oct4, Nestin, Lgr5, Sox2, CD24 were assessed by western blotting. HSP90 expression was assessed by western blotting and immunohistochemistry (IHC). Glucose consumption, lactic acid production and ATP levels were measured using commercially available kits. Extracellular acidification rates (ECAR) were measured using the Seahorse XFe/XF analyzer.

**Results:**

HSP90 was upregulated in spheroid cells compared to parental cells. HSP90 facilitated the characteristics of SCs through enhancing self-renewal capacity, glucose consumption, lactic acid production, total ATP, ECAR and glycolysis. 2-DG, an inhibitor of glycolysis, reduced HSP90 expression and inhibited the stemness of glioma cells.

**Conclusions:**

We show that HSP90 accelerates stemness and enhances glycolysis in glioma cells. Inhibition of glycolysis with 2DG prevented stemness. This reveals new roles for HSP90 during glioma progression and highlights this protein as a potential target for much-needed anti-glioma therapeutics.

## Background

Gliomas are the most common tumors of the central nervous system (CNS), with high morbidity and mortality [1–3] [4]. The typical features of gliomas include angiogenesis, aggressive growth and necrosis. Based on pathological assessments, the World Health Organization (WHO) criteria classifies gliomas as Grade I-II (astrocytomas, oligodendrogliomas, pleomorphic xanthoastrocytomas, and certain ependymomas), highly malignant Grade III (anaplastic astrocytomas, anaplastic oligodendrogliomas, and anaplastic ependymoma) and Grade IV (glioblastoma, GBM) [5, 6]. GBM represents the most malignant and familiar type, accounting for ~ 45% of all gliomas. Despite advances in cancer diagnosis and therapeutics, the 5-year survival rates of GBMs remain extremely low [7]. Current treatment regiments include surgical resection, radiotherapy and chemotherapy, but prognosis is low [8, 9]. More effective treatment strategies are urgently required.

Cancer stem cells (CSCs) promote tumor development in head and neck cancer [10, 11] [12], breast cancer [13], gastric cancer [14] and glioma. At the molecular level, miR-663a [15], the FOSL1-miR-27a-5p axis [16], and miR-504 regulate the stemness of Glioma CSCs, influencing both tumor progression and radiosensitivty [17]. Heat shock protein 90 (HSP90) is a molecular chaperone that is frequently overexpressed in gliomas. HSP90 regulates the assembly and folding of nascent polypeptides and has been proposed to enhance the adaptation capacity of glioma cells to stress conditions, promoting tumor growth.

A role for HSP90 in the stemness of glioma cells has not been described. Here, to further our understanding of the role of HSP90 in tumor development, we investigated its role in enhancing self-renewal capacity, glucose consumption, lactic acid production, total ATP, ECAR and glycolysis in spherical cell culture as an effective strategy to form CSCs.

## Methods

All experimental protocols were approved by the Ethics Committee of Beijing Tiantan Hospital (VS212601442) and were performed in accordance with relevant guidelines and regulations.

### Cell culture

The glioma cell lines U87MG-SLC and U251 were purchased from American Type Culture Collection (ATCC). Cells were cultured in Dulbecco’s modified Eagle medium (DMEM) supplemented with 10% fetal bovine serum (FBS) in a humidified chamber at 37 °C with 5% CO_2_.

### Self-renewal assays

Self-renewal capacity was assessed through spheroid-formation. Cells were seeded into 24-well plates and cultured in DMEM supplemented with 0.8% methylcellulose (Sigma), B27 (1:50), Epidermal growth factor (EGF) (20 ng/mL), basic fibroblast growth factor (bFGF) (20 ng/mL) and LIF (10 ng/mL). Cells were cultured for 1-2 weeks at 37 °C in 5% CO_2._ Spheroids were imaged under a light microscope.

### Glucose consumption, lactate production and ATP detection

Commercial glucose and sucrose assay kits (Sigma-Aldrich, MAK013) were used to measure glucose consumption in the culture media. Lactate Colorimetric Assay Kit II (Biovision, K627-100) was used to assay lactic acid levels. Commercial kits (Nanjing Jiancheng) were used to detect total ATP.

### Glycolysis level analysis

Cells were seeded into Seahorse XF96 plates and treated with glucose, oligomycin, and 2-deoxy-d-glucose (2-DG). Cells were then loaded into a hydrated sensor cartridge at appropriate ports and glycolysis stress measured on a Seahorse XFe/XF Analyzer.

### Chemo sensitivity assays

Cells were treated with 0-100 μM temozolomide (TMZ, Sigma-Aldrich, USA) for 72 h and cell viability evaluated using cell counting kit-8 (CCK-8) kits. IC50s were determined by measuring the absorbance at 450 nm using a microplate reader (Bio-rad, USA).

### In vivo xenograft nude mouse models

Male BALB/c nude mice (4-5 weeks old, total *n =* 72) were purchased from Charles River (Beijing) and subcutaneously injected with 1 × 10^6^ glioma cells (*n =* 6 in each group). Tumor sizes were recorded every 7 days. After 1 month, mice were sacrificed and tumor volumes and weights were recorded. All mice survived the procedure. All animal experiments were performed in accordance with ARRIVE guidelines (https://arriveguidelines.org).

### Transwell assays

Transwell chambers with 8-μm pores (Corning, Inc.) were precoated with (or without) matrigel for the assessment of cell invasion or migration, respectively. Cells (200 μL) in serum-free media were added to the upper chambers, whilst 600 μL of medium containing 20% FBS was added to the lower chamber. After 48 h, invading or migrating cells were fixed in 4% paraformaldehyde, stained with 0.1% crystal violet, and counted on an inverted microscope.

### Western blotting

Cells were lysed in RIPA buffer and proteins were resolved on SDS-PAGE gels and transferred to polyvinylidene fluoride (PVDF) membranes (Amersham, USA). Membranes were blocked in non-fat milk and probed with anti-HSP90 (1:1000, ab13492, Abcam, Shanghai, China), A2B5 (1:1000, ab53521), CD133 (1:1000, ab222782), Nestin (1:1000, ab105389), Sox2 (1:1000, ab97959) and GAPDH (1:1000, ab8245) primary antibodies. Membranes were labeled with HRP-anti-rabbit IgG (ab6721, 1:2000, Abcam) secondary antibodies and proteins were detected using the enhanced chemiluminescence system (ECL, ThermoFisher, USA).

### Immunohistochemistry (IHC)

Paraffin-embedded tumor tissues from nude mice were sectioned and probed with primary anti-HSP90 antibodies overnight at 4 °C. After washing with PBS, sections were labeled with secondary antibodies and imaged under a light microscope (Olympus, Tokyo, Japan).

### Statistical analyses

Data represent the mean ± standard deviation (SD) of at least three independent experiments. Statistical analysis was performed using SPSS 22.0 software. Single group comparisons were performed using a Student’s t-test. Multiple group comparisons were performed using a one-way ANOVA followed by Tukey’s post hoc test. *P <* 0.05 was deemed statistically significant.

## Results

### HSP90 is upregulated in spheroid cells

**Fig. 1:**
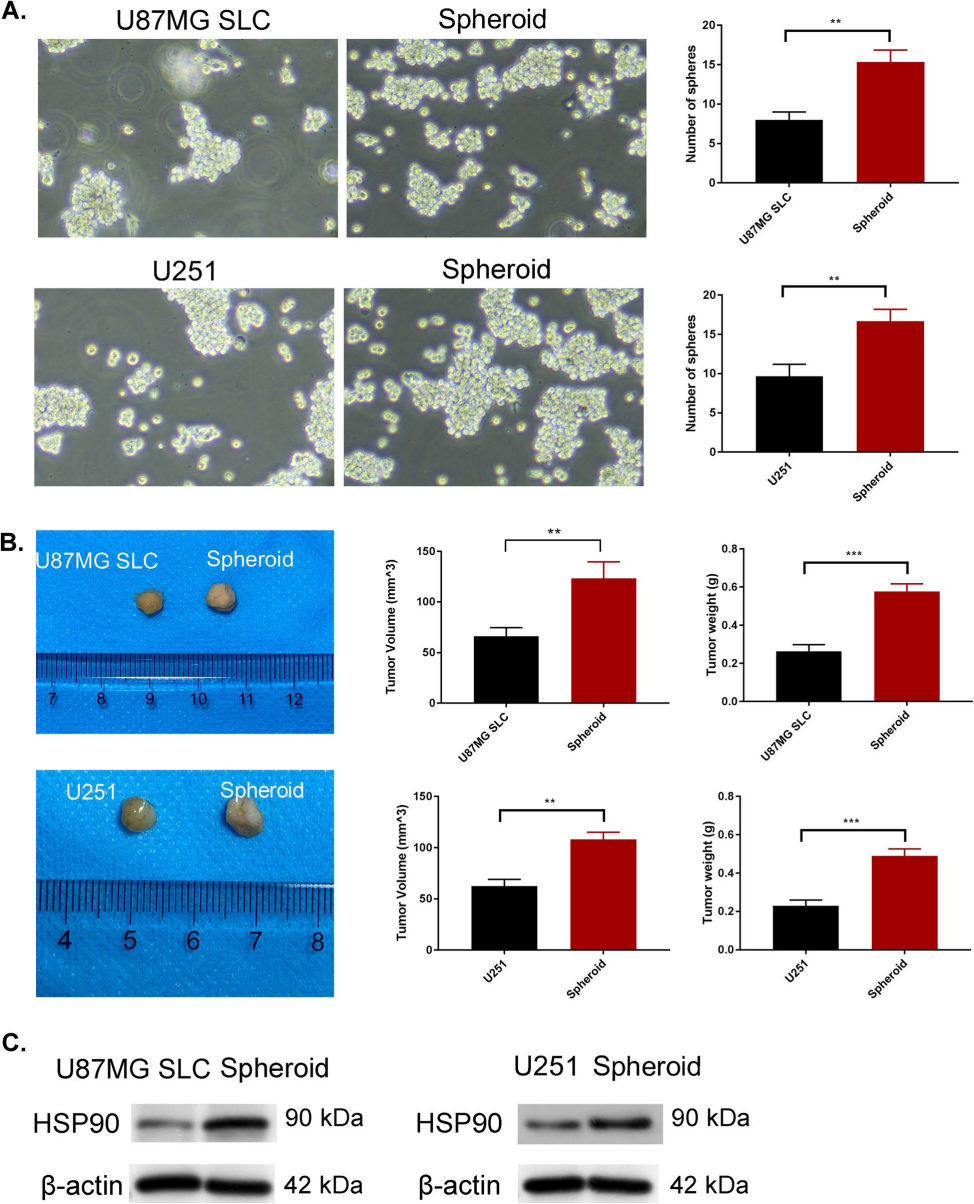
Heat shock protein 90 (HSP90) expression is upregulated in spheroid cells. **A** Stemness of glioma cells (U87MG SLC and U251 cell lines) assessed through self-renewal assays. **B** Evaluation of tumor growth (size, volume and weight) using nude mice in vivo. *n =* 6 mice in each group. **C** Expression of HSP90 detected through western blotting. ^**^p < 0.01

Spheroid-forming cultures of U87MG and U251 cells was produced to enrich glioma CSCs. After 1-week, non-adherent spheres containing 40 to 100 cells formed and were termed “spheroids”. All experiments were performed using third-passage spherical cells. Self-renewal assays showed an increased number of colonies in the spheroid group (*p <* 0.01) (Fig. 1A), indicative of higher self-renewal capacity of compared to parental cells.

A xenograft model was used to determine the tumorigenicity of the spheroids. Compared to parental cells, the tumorigenic ability of the spheroids was enhanced (Fig. 1B). Tumor volumes were also higher in the spheroids compared to parental U87 and U251 cells (Fig. 1B). Upon assessment of the expression of HSP90 in spheroids, a marked increase was observed compared to parental U87MG and U251 cells (Fig. 1C). This was suggestive of a potential role for HSP90 in the stemness of glioma cells.

### HSP90 influences the stemness of glioma cells

**Fig. 2:**
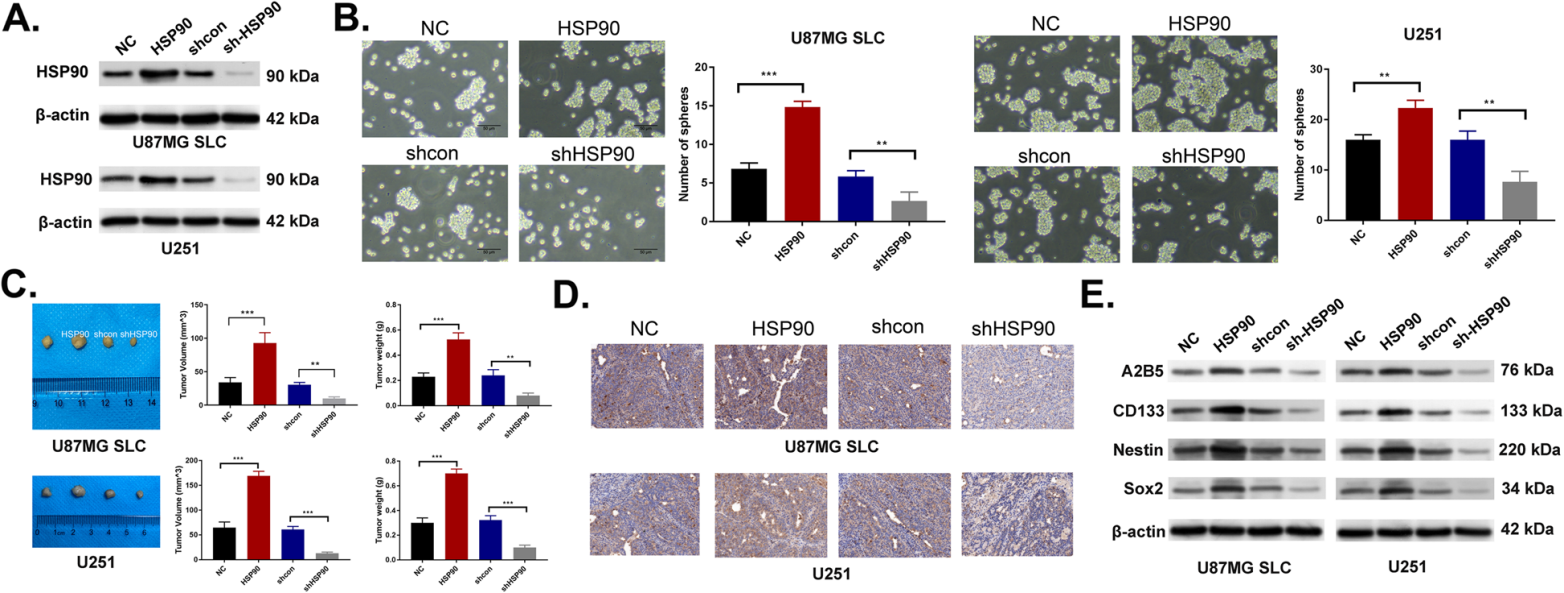
HSP90 influences the stemness of glioma cells. **A** HSP90 expression in oe-HSP90 and sh-HSP90 cells was evaluated by western blotting **B** Assessment of the stemness of glioma cells after overexpressing or inhibiting HSP90 through self-renewal assays. **C** Tumor growth evaluation (size, volume and weight) in HSP90 overexpressing or silenced nude mice in vivo assay. *n =* 6 mice in each group. **D** Determination of HSP90 expression in IHC assays. **E** Levels of A2B5, CD133, Nestin, and Sox2 detected through western blotting. ^**^p < 0.01, ^***^p < 0.001. NC: negative control, HSP90: HSP90 overexpression, shcon: shRNA control, shHSP90: shRNA HSP90

To further investigate the influence of HSP90 on stemness, retroviral transduction was used to stably overexpress (oe-HSP90) or knockdown (sh-HSP90) HSP90 in U87MG and U251 cells. Successful overexpression and silencing were confirmed by western blotting and IHC (Fig. 2A & D). The self-renewal capacity of cells was strengthened after overexpressing HSP90 and weakened following its silencing (Fig. 2B). Additionally, tumors were larger in size, volume and weight in the oe-HSP90 group, compared to the smaller size, volume and weight of sh-HSP90 tumors (Fig. 2C). The levels of A2B5, CD133, Nestin, and Sox2 were up-regulated following HSP90 overexpression and downregulated following HSP90 suppression (Fig. 2E). These data confirm a role for HSP90 in the stemness of glioma cells.

### HSP90 enhances glucose consumption and lactic acid production in glioma cells

**Fig. 3:**
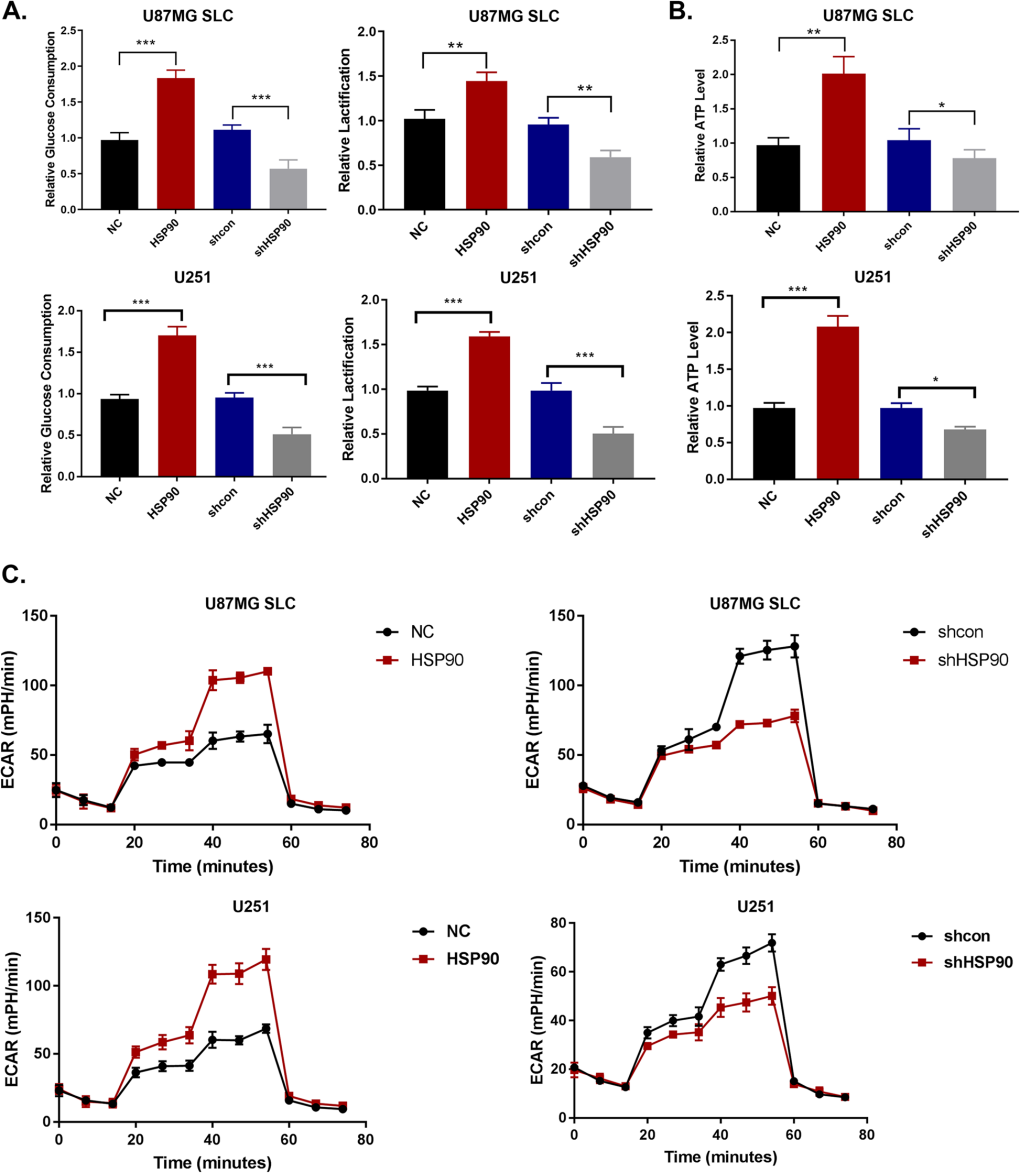
HSP90 enhances glucose consumption and lactic acid production of glioma cells. **A** Assessment of glucose consumption and lactic acid production in U87MG SLC and U251 cells. **B** ATP assessments. **C** ECAR detected on a Seahorse XFe/XF Analyzer. ^*^p < 0.05, ^**^p < 0.01, ^***^p < 0.001. NC: negative control, HSP90: HSP90 overexpression, shcon: shRNA control, shHSP90: shRNA HSP90

We next investigated whether HSP90 affects glycolysis in Glioma spheroids. Relative glucose consumption and lactic acid production were modestly enhanced following the overexpression of HSP90 and reduced following its silencing in U87 and U251 cells (Fig. 3A). Additionally, total ATP levels modestly increased following HSP90 overexpression and declined following HSP90 silencing (Fig. 3B). To further verify the effects of HSP90 on glycolytic metabolism, extracellular acidification rates (ECAR) were measured. ECAR levels increased in oe-HSP90 cells and declined in sh-HSP90 cells (Fig. 3C). These data suggest a role for HSP90 in the enhanced glucose consumption and lactic acid production of glioma cells.

### 2-DG treatment inhibits the stemness of glioma cells through the suppression of glycolysis

**Fig. 4:**
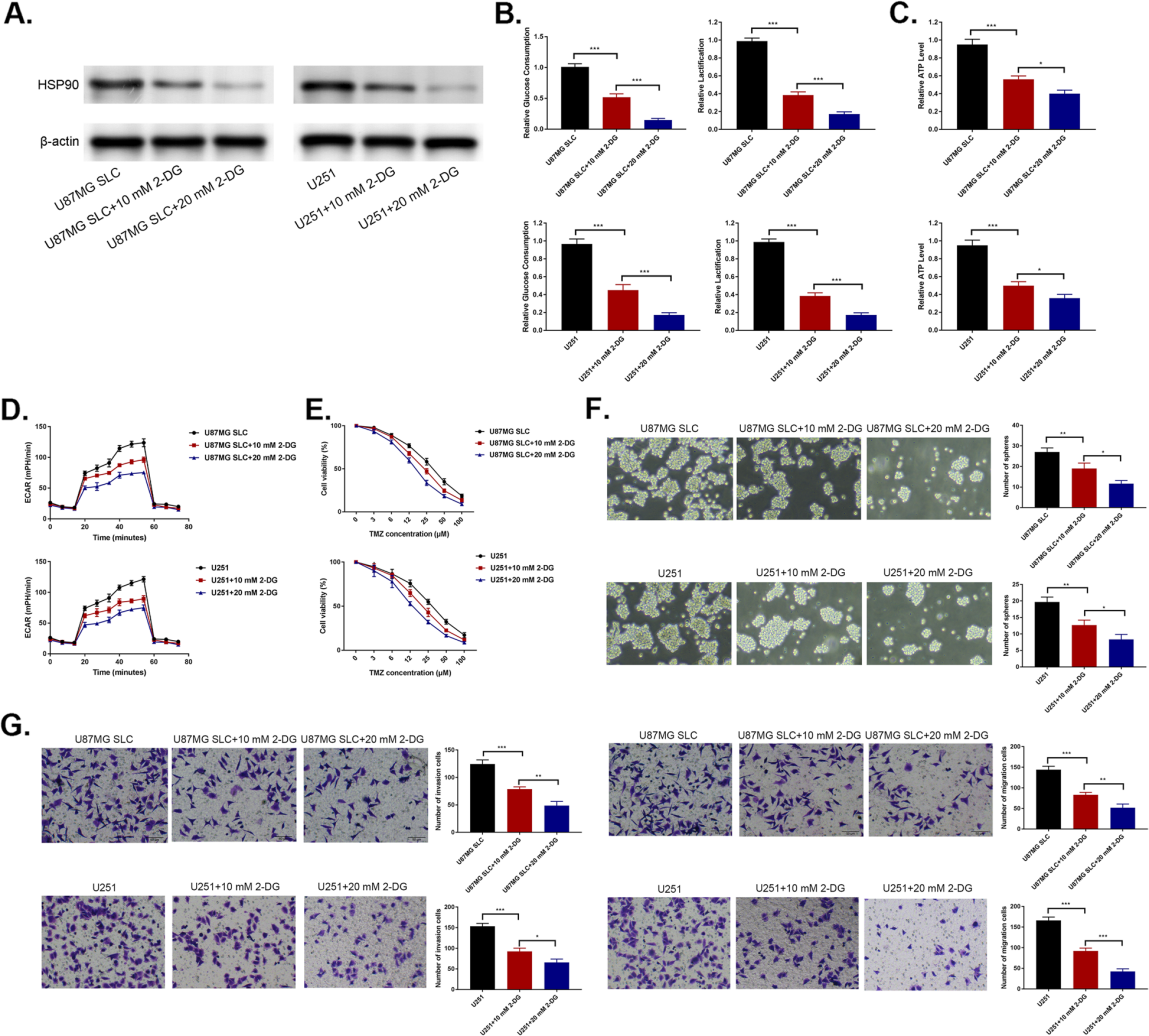
2-DG treatment inhibits the stemness of glioma cells through the suppression of glycolysis. Cells were treated with 10-20 mM 2-DG. **A** Assessment of glucose consumption and lactic acid production. **B** Total ATP levels. **C** ECAR. **D** Stemness through self-renewal assays. **E** Migration and invasion through Transwell assays. **F** IC50 (half maximal inhibitory concentration) through CCK-8 assays. ^*^p < 0.05, ^**^p < 0.01, ^***^p < 0.001

To determine whether CSC-like characteristics are influenced by glycolysis, U87 and U251 cells were treated with the glycolytic inhibitor, 2-DG. Treatment with 2-DG (10-20 mM) led to marked downregulation of HSP90 (Fig. 4A) and decreased glucose consumption and lactic acid production (Fig. 4B). ATP levels and ECAR also decreased following 2-DG treatment (10 or 20 mM) (Fig. 4C-D). Moreover, the capacity for self-renewal was weakened by 2-DG (Fig. 4E), as was the migration and invasive capacity of both cell lines (Fig. 4F). The sensitivity of cells to TMZ also dramatically increased following 2-DG treatment (Fig. 4G). Collectively, these findings indicate that the inhibition of glycolysis suppresses the stemness of enhances the chemosensitivity of glioma cells.

## Discussion

Several reports have confirmed the existence and significance of CSCs in glioma [18, 19]. CSCs are tumor cells that possess the characteristics of self-renewal, multiple differentiation, high tumorigenicity and drug resistance [20]. Stem cell markers such as CD44, A2B5, Oct4, Nestin, Lgr5, Sox2, CD24, and CD133 regulate CSCs and can be used as markers of their activity. Spherical cell culture is an effective strategy to form CSCs [21]. In this study, glioma CSCs were successfully produced using this method.

HSP90 is a well-characterized chaperone in eukaryotes, regulating various cellular processes including signal transduction, protein folding, protein degradation and the maturation of client proteins [22, 23]. Recent evidence shows that HSP90 regulates the function of over 200 proteins and plays a key role in oncogenesis [24]. Of note, HSP90 is active in tumor cells but largely dormant in healthy cells. The expression of HSP90 is also 2-10 fold higher in tumor cells [25, 26]. HSP90 is necessary for malignant transformation and represents a potential target for cancer treatment. Here, we showed that HSP90 is upregulated in spheroid cells compared to the parental cells and facilitates stem cell-like characteristics, including self-renewal capacity and the tumorigenicity of glioma cells.

Several studies have investigated the association between stemness and glycolysis in cancer. Increased glycolysis accelerates the stemness and EMT phenotypes of gemcitabine-resistant pancreatic cancer cells [27]. Sodium lactate mediates KDM6B mediated glycolytic metabolism to facilitate human mesenchymal stemness [28]. ETV4 activates glycolysis to strengthen breast cancer cell stemness [29]. CD44ICD regulates PFKFB4-mediated glucose metabolism to facilitate breast cancer stemness [30]. HSP90 has been shown to facilitate glycolysis and cell proliferation through Thr-328 phosphorylation in hepatocellular carcinoma [31]. Here, we investigated whether HSP90 affects the stemness of glioma cells through its effects on glycolysis. We found that HSP90 enhanced glucose consumption, lactic acid production, total ATP and ECAR levels, thereby promoting glycolysis. 2-DG, an inhibitor of glycolysis inhibited the stemness of glioma cells, suppressed HSP90 expression and enhanced chemosensiitivty to TMZ. These data are suggestive of a potential role for HSP90 in glycolysis driven stemness. This now requires confirmation in patient derived glioma SCs as a more physiological model of the disease phenotype.

## Conclusions

In summary, we show that HSP90 facilitates stemness and enhances glycolysis of glioma cells. We also show that the inhibition of glycolysis suppresses HSP90 expression and Glioma stemness, which may reveal a new function of HSP90 in glioma, although this requires further clarification. Some limitations of this study should be noted, including the lack of an orthotropic model or patient derived Glioma samples for the confirmation of our findings. Other cancer phenotypes including autophagy, angiogenesis, exosome production and immune responses could also be investigated in response to HSP90 overexpression or silencing, to fully reveal its role in all aspects of glioma progression.

## Data Availability

All data generated or analyzed during this study are included in this published article.

## Abbreviations

SCs: Stem cells
HSP90: Heat shock protein 90
